# Experimental characterization of colloidal silica gel for water conformance control in oil reservoirs

**DOI:** 10.1038/s41598-022-13035-1

**Published:** 2022-06-10

**Authors:** Zahra Ghaffari, Hosein Rezvani, Ali Khalilnezhad, Farid B. Cortes, Masoud Riazi

**Affiliations:** 1 Enhanced Oil Recovery (EOR) Research Centre, IOR/EOR Research Institute, Shiraz University, Shiraz, Iran; 2 Faculty of Petroleum and Natural Gas Engineering, Sahand University of Technology, Tabriz, Iran; 3 Department of Chemistry, University of Hull, Hull, UK; 4 Grupo de Investigación en Fenómenos de Superficie-Michael Polanyi, Departamento de Procesos y Energía, Facultad de Minas, Universidad Nacional de Colombia, Sede Medellín, 050034 Medellín, Colombia; 5 Department of Petroleum Engineering, School of Chemical and Petroleum Engineering, Shiraz University, Shiraz, Iran

**Keywords:** Crude oil, Energy, Chemical physics, Nanoparticles

## Abstract

High water production in oil fields is an area of concern due to economic issues and borehole/wellhead damages. Colloidal gels can be a good alternative to polymers to address this as they can tolerate harsh oil reservoir conditions. A series of bottle tests with different silica and NaCl concentrations were first conducted. The gelation time, cation valence, rheology, and viscosity were investigated to characterize the gels. The applicability of solid gels in porous media was finally inspected in a dual-patterned glass micromodel. Bottle test results showed that increasing NaCl concentration at a constant silica concentration can convert solid gels into two-phase gels and then viscous suspensions. Na^+^ replacement with Mg^2+^ resulted a distinctive behaviour probably due to higher coagulating ability of Mg^2+^. Rheology and viscosity results agreed with gelation times: gel with shortest gelation time had the highest viscosity and storage/loss modulus but was not the most elastic one. Water injection into glass micromodel half-saturated with crude oil and solid gel proved that the gel is strong against pressure gradients applied by injected phase which is promising for water conformance controls. The diverted injected phase recorded an oil recovery of 53% which was not feasible without blocking the water zone.

## Introduction

Water conning from an underneath strong aquifer or water channeling during an aqueous phase injection into fractured/faulted oil reservoirs can increase water production in oil wells^1^. High water cuts usually pose severe problems in oil fields: depositing a layer of salts on pipelines and thus corrosion of overhead equipment, high separation costs, and high energy usage^2^. Even worse, the produced water might be incredibly high that the oil production from the well comes to an end for economic reasons. The main reasons for the production of excess water are variations in permeability and large viscosity differences between reservoir fluids^1^. To address the issue, numerous mechanical (e.g. plugging water producer zone) and chemical solutions have been introduced^3^. The latter mostly includes treatments by polymers and gels to block the water-bearing zones. Recently, the application of oil in water emulsions and foams for conformance control to reduce the fluid flow of the high permeability regions in oil reservoirs has been suggested^4–6^.

Gels are systems of a liquid and a chemical, e.g., polymers or colloidal particles, capable of showing both liquid- and solid-like behaviours. Generally, a polymer gel is a 3D network of a crosslinked polymer swollen by a solvent like water^7^. Polymer gels are formed due to the polymerization by chemical reactions. The nature/kinetics of the formed chemical bonds are determining in the resulting gel features. In polymer gels, the gelation volume is controlled by the degree of polymerization. In other words, the hydrolysis and related polymerization reactions influence the gel characteristics and the resulting inorganic material^8^.

Colloidal dispersions are particles dispersed in a liquid phase. When interparticle attractions sufficiently exist e.g. by the addition of salt or very high particle loading, particle collisions due to the Brownian motion increase which causes the particles to irreversibly aggregate in the liquid and create particle strands. The resulting material can be progressively changing to a 3D structure, i.e. an interconnecting network of particle strands, which is known as colloidal gels^9–11^. There are several factors governing the extent of aggregation in a dispersion: temperature elevation, pH reduction, surface charge screening by addition of salts, and increasing particle loading^12,13^. In case that the attractive forces between particles are high, the interparticle bonds become strong and create fractal clusters. Although liquid has the larger weight in colloidal gels, its solid-like behaviour results from the network of particles in the dispersant which causes the gel to benefit from some unique properties, e.g. non-zero yield stress^14^. The particle network here acts as cross linking which determines the final gel structure and hardness. The attractive forces responsible for colloidal gel formation are mostly short-range forces which can be generated by altering the particle surface charges, screening of interparticle repulsions, and depletion interactions originating from dissolved forces which are not adsorbed, bridging like polymers, and a change in solvent properties^11^.

Several mechanisms and theories have been proposed to describe the gelation by colloidal particles. Campbell et al.^15^ studied the colloidal gelation by 3D fluorescence confocal microscopy. They resulted that when the packing fraction is small, particle clusters with relative stability are formed however at high packing fractions the clusters become bigger until the interconnecting network of particles are formed for gelation. They depicted the colloidal gel as tetrahedral clusters of particles connected to each other like compact particle chains. By experimenting light scattering and rheology, Manley et al.^16^ stated that strength of colloidal silica gels is time dependent. The elastic moduli rise with time regardless of particle concentration. They concluded that locally increased elasticity of bonds between adjacent particles are responsible for forming particle network and gelation. Smith and Zukoski^17^ studied the kinetics of colloidal fumed silica gel in ethanol. They pointed out that the gelation of particles at low concentrations is mainly due to the particle aggregations at the primary minimum of the potential energy curve by the formation of hydrogen bonds and dispersion forces. The solvation forces developed between particles induces an energy barrier which makes the gelation process in this situation slow. The secondary minimum, created by solvation forces and London dispersion attractions, indeed limits the diffusion of particles at large distances at a critical particle loading.

The specialized literature on using colloidal particle gels for water conformance control in oil reservoirs is scarce. In one of the very first studies in 1991, Jurinak and Summers^18^ studied 7 nm Ludox silica (6–15 wt%) gelled with NaCl for water conformance control. In lab scale, they observed a permeability reduction from 10–500 to 0.01 md for consolidated cores and from 1–3 to 0.5–1.0 d for unconsolidated sand packs after saturating with colloidal silica gel. They reported that the gel in consolidated cores can tolerate a pressure gradient of 2500 psi/ft. In field scale, they reported that previously hydraulically fractured regions were prosperously blocked by colloidal silica gel with long-term stability. Liang et al.^19^ used different gel formulations including resorcinol–formaldehyde, chloride-xanthan, acetate-polyacrylamide, and colloidal silica for water conformance control in sandstone cores by measuring oil/water permeabilities before and after treatments with gels. The results indicated that almost all gels except to colloidal gel reduced water permeability more than oil. In case of colloidal silica gel, the oil and water relative permeability were reduced to about the same value (i.e. the same oil and water residual resistance factor). They observed a breakdown in colloidal gel with pressure gradients < 200 psi/ft which is in contradiction to that reported by Jurinak and Summers^18^. Both studies have used a high particle concentration.

As stated, there are numerous methods to address conformance control in oil reservoirs. As compared with an aqueous phase e.g. brine, emulsified or foamed solutions has a higher ability in blocking high permeable zones to divert the flow toward other areas to produce oil. However, these methods are more local with short-term effects and their success highly depends on foam/emulsion stability within reservoirs. Environmentally hazardous, expensive polymer gels have been long proposed for water shut-off applications, but they are mostly degradable at high temperature/salinity conditions of oil reservoirs which limits their applications. Viscous polymer solutions may be also challenging when it comes to injecting into the reservoirs^20^. However, cheap solid particles are mostly mined from naturally occurring ores. This removes the environmental concerns about them. They can also withstand high salinity, pressure, and temperature of oil reservoirs (e.g. silica has a typical melting point of ca. 1550 °C^21^). Injectivity problems are also absent for the injection of dispersions (as the gellant of colloidal gels) with a viscosity comparable to water. In the current study, the application of colloidal silica gel is studied as a promising solution for water shut-off. In addition to the low cost and unique physical properties, silica particles can be also used as a grouting material that can penetrate and seal fractures^22,23^. A series of bottle tests were first performed to determine the gelation regions formed by lower concentrations of silica and a wide range of salinities (i.e. formation brine salinity), as compared to previous studies. Machine learning using CARET in R was used to model the bottle test results. The gels were then characterized by gelation time, rheology, and viscosity. To investigate the efficiency of solid gels in porous media for water conformance control purposes in oil reservoirs, a novel dual-patterned glass micromodel was designed and constructed by the research group, which is to the best knowledge of the authors, has not been used before. The porous media mimics a water-producing zone topped with an oil layer which is required to be blocked otherwise natural production comes with unfavoured high water production and an EOR method might fail.

## Materials and methods

### Materials

Aqueous hydrophilic silica (30 wt%) with a SiO_2_/Na_2_O ratio of 75–100 was used in this study. The bluish stock dispersion had a pH of 9–10 with particles of 10–30 nm (averagely 20 nm). Sodium chloride (NaCl) and magnesium chloride hexahydrate (MgCl_2_.6H_2_O) salts both of ACS reagent grade (purity ≥ 99%) were purchased from Sigma Aldrich (United States of America, USA) and used to adjust the salinity of dispersions. Crude oil was obtained from one of the Iranian oil reservoirs with high water cut problems. The properties of crude oil are presented in Table 1.

**Table 1:** Specifications of crude oil.

Specification	Amount	Unit
Viscosity at 22 °C	8.3	mPa s
Density at 22 °C	0.85	g/cm^3^
Gravity	35.4	°API
Total acid number at 26 °C	0.15	mgKOH/g
Total basic number at 26 °C	1.52	mgKOH/g

### Methods

#### Determination of gelation regions

Different concentrations of silica (1–6 wt%) were diluted from the stock 30 wt.% dispersion by deionized water (DIW) followed by the addition of different concentrations of NaCl (1–11 wt%). The mixture was stirred for 30 min at room temperature (20–25 °C) on a magnetic stirrer. The pH of all dispersions was measured using 86502 AZ pH meter (AZ Instrument Corp., Taiwan). The dispersions were then moved to glass vials with screw caps and monitored with time for 24 h at room temperature to record any visual change in the appearance by picturing technique. Gelation of the dispersions was tested by rotation of sample vials by 180° (i.e., upside down). The rotation was performed gently in order not to disturb the equilibrium.

#### Gelation time

The gelation time of solid gels was determined by both bottle tests and UV–Vis spectroscopy. Dynamica DB-20S spectrophotometer (UK) was used to measure the absorbance of particles. It is noteworthy to mention that the gelation time determined in bottle tests are the time of complete solid gelation whereas the one measured using spectroscopy is the time of the start of gelation (see Supporting information for details). Fresh dispersions forming a single-phase solid gel were prepared using similar preparation steps (e.g., stirring time). The absorbance of dispersions was measured with time at 400 nm. The start time of gelation was detected in the plot of absorbance versus time where the diagram begins to level off after an increase.

#### Effect of type of salt on gelation

To investigate the effect of valence of cation on the gelation, sodium chloride was replaced by magnesium chloride in bottle tests performed in section “Determination of gelation regions” for some random points and similarly visual changes were recorded with time.

#### Rheology of gels

Completel solid gels spotted in bottle tests were analysed rheologically by Anton Paar (UK) and cone/plate method at 25 °C. Fresh gellants (i.e., dispersions forming gels after 24 h) were prepared using the same concentrations of silica and salt and left for 24 h at room temperature with no movement to ensure that the gelation is not disturbed during visual inspection. The viscosity, storage modulus (G′) and loss modulus (G″) of solid gels were measured at different shear strains/stresses.

#### Displacement study by glass micromodel

**Figure 1:**
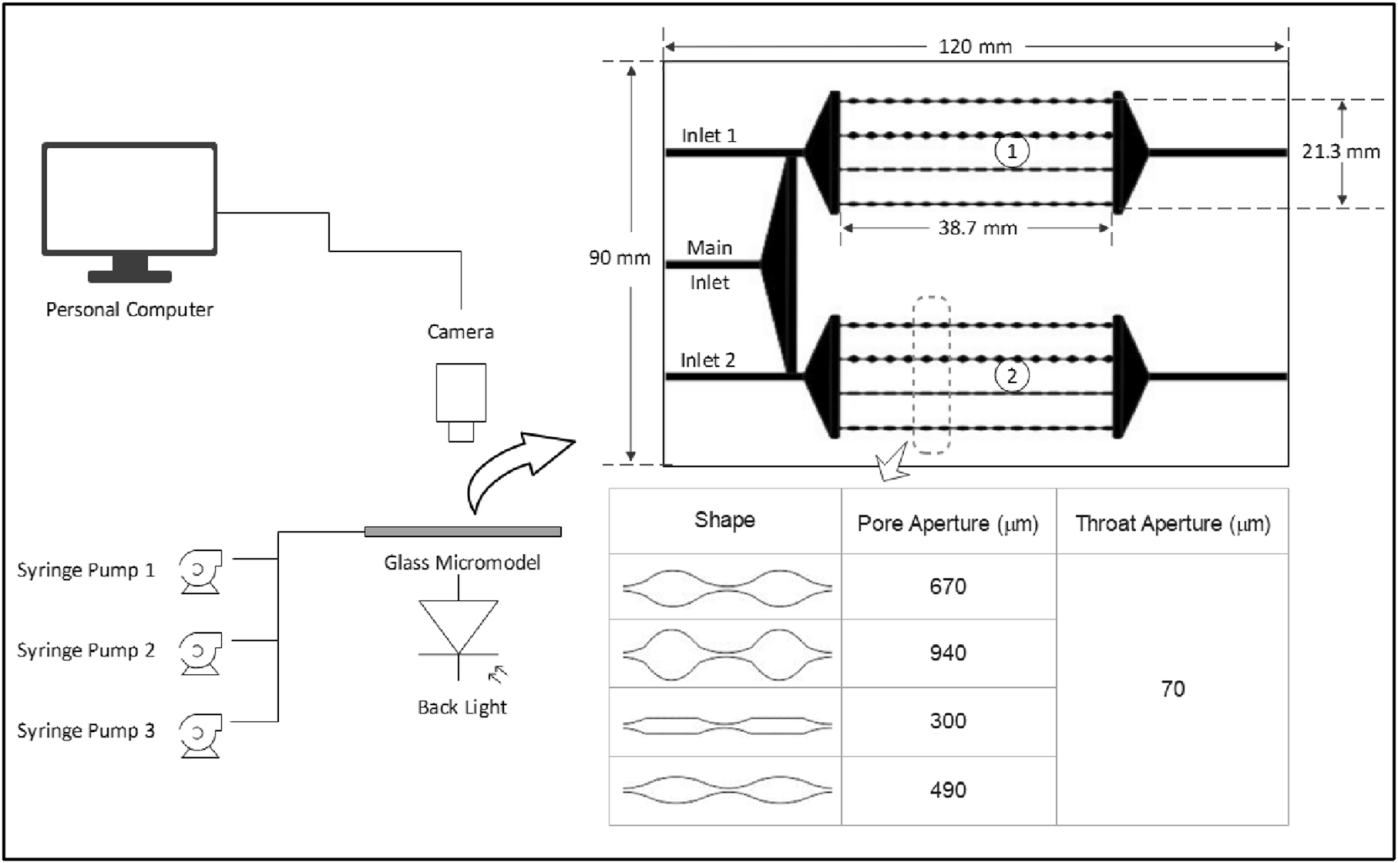
Setup of displacement study in glass micromodel.

To investigate the efficiency of solid gels in porous media for water conformance control purposes in oil reservoirs, a novel double-patterned glass micromodel was designed and constructed by the research group, which is to the best knowledge of the authors, has not been used before. Figure 1 shows the setup of the displacement study. As shown, the setup includes three syringe pumps (LA-30, LANDGRAVE HLL, Germany), a back light source, a glass micromodel, and a camera connected to a computer. The pattern of the glass micromodel is consisted of two identical porous media 1 and 2 with two separate inlets 1 and 2. Each porous medium is consisted of four parallel tubes which are different in pore size but similar in throat size (200 µm) and depth (150 µm). The two porous media are exactly similar in physical properties, i.e., size of pore throats, length, inlet shape, channel depth, pore volume (PV), and porosity. The separate inlets (inlets 1 and 2) facilitate a single-phase saturation of crude oil or the aqueous phase. A main inlet connects to the two porous media and used for the main injection. The PV and the porosity of each porous medium excluding the inlets are 14.8 cm^3^ and 12%, respectively. Injection rate in all experiments was 0.1 mL/min. Such a design of micromodel mimics the water bearing zones topped by oil layers in oil reservoirs. The reason for this design is first to question whether or not the gel formed in porous medium 2 is strong enough against pressure gradients applied by the injected phase (through the main inlet) to block porous medium 2. If the gel is not displaced, it must be observed to be diverting the flow of the injected phase toward the oil-saturated zone (porous medium 1) for potential EOR.

##### Control experiment

Prior to the main experiment, the designed glass micromodel was tested to ensure the functionality of the two porous media, inlets, and outlets. The horizontal glass micromodel was fully saturated with crude oil and rested for 24 h at the room temperature. DIW was then injected into the glass micromodel via the main inlet for 2 PVs with an injection rate of 0.05 mL/min. As all parts, i.e. the two patterns, inlets, and outlets are identical, it is expected that nearly the same amount of oil is displaced from the two porous media with DIW. During flooding, different parts of the micromodel were monitored with camera to inspect whether the two parts work similarly.

##### Main experiment

A dispersion forming a solid gel after 24 h at room temperature based on bottle tests was chosen as a candidate for saturating porous medium 2 in the glass micromodel. Following blocking the main inlet using a rubber stopper to reduce air in the glass micromodel, crude oil and the fresh dispersion were separately injected into the two porous media simultaneously. During saturation process, the glass micromodel was placed horizontally on a flat surface. After saturation, all inlets and outlets were blocked, and the micromodel was allowed a resting time of 24 h at room temperature for the gel to be formed from the dispersion in the porous medium and for the crude oil to equilibrate with glass surface (i.e., to impart some extent of oilwetness in porous medium). Extensive care was given to the setup not to be moved so that not disturb the system.

For the main purpose of this experiment, i.e., investigation of the strength of the formed gel in porous media for water conformance control, DIW was injected through the main inlet into the micromodel. During the main injection, inlets 1 and 2 were blocked using rubber stoppers. Macro- and microscopy were used to analyse the fluid displacement during the experiment. The amount of oil recovered in this part was determined using comparing the black pixels (i.e., residual oil) of the porous medium image with the initial status when it is completely saturated with oil (i.e., all black pixels) using image analysis technique.

## Results and discussions

### Determination of gelation regions

**Figure 2:**
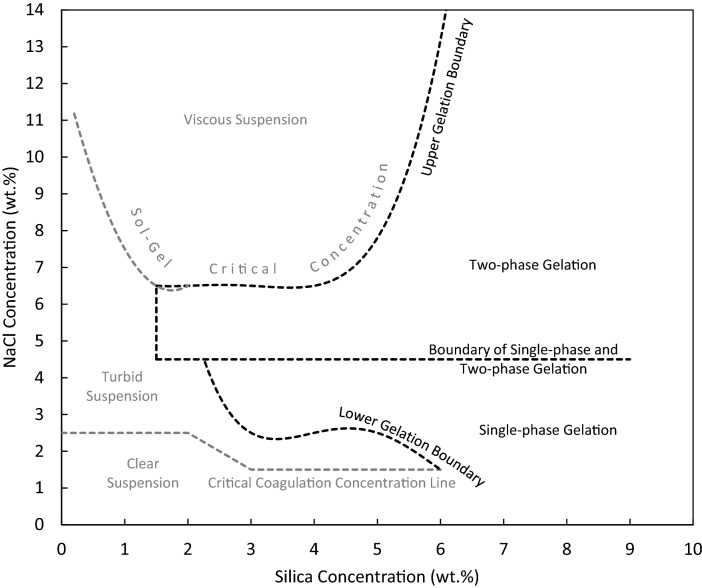
General map of bottle test results based on the experimental observations in bottle tests. The curves and lines are fits to the data.

Figure S-1 (See Supporting Information) confirms that particles alone (no salt) cannot form gel at the concentration range used in this study, and the gelation is purely related to the effect of added ions on the interactions between particles. Figure S-2 shows the fate of different dispersions containing different concentrations of silica and NaCl in DIW placed at room temperature for 24 h. These results have been mapped onto the plot in Fig. 2. This figure provides a comprehensive map of gelation with relative to the silica and salt concentrations. The boundaries plotted in this figure are fitted models (R^2^ > 0.8) to the data presented in Fig. S-2.

There are five potential states based on the concentrations of particles and NaCl in Fig. 2: clear suspension, turbid suspension, viscous suspension, single-phase solid gel, and two-phase gel (gel + liquid). Figure S-3 (see Supporting Information) shows a typical photo of different classes obtained after 24 h in glass vials. Briefly, clear suspensions are those with no visual change in colour with the same bluish colour as observed initially. Turbid suspensions represent dispersions with some extents of aggregation. Viscous suspensions are milky and viscous. Single-phase gels are completely solid gels with no liquid while two-phase gels are solid gels topped with a liquid. For completely solid gels, the gel does not flow on rotating the vials. In a study by Metin et al.^24^, it was resulted that two-phase gelation occurs at low silica concentration whereas a single-phase gel is formed at high particle concentration, which is in contrast with the findings of this study. Figure 2 depicts that the single- and two-phase gelation is dependent on the salt concentration, not particle concentration. For silica concentrations above 2 wt%, two-phase gelation has mainly occurred for 5 and 6 wt% salt.

Critical coagulation concentration (CCC) is the concentration of salt above which the dispersion becomes unstable. By adding salts to a dispersion, above the CCC, bare charged particles of the dispersion start to aggregate due to the screening of the particle surface charges by ions which results in an unstable dispersion. With an increase in salt concentration, the energy barrier in the total interaction energy curve decreases until it becomes zero for an unstable dispersion^25^. Above CCC of the salt, colloidal particles can form gels. The formed gels are produced by entrapment of particles. At large distances, the electrostatic repulsion between particles exceeds the Van der Waals (VdW) attractions. However, at short distances it might be defeated. At short distances, VdW attractions aggregate particles into larger 2D aggregates, which can be controlled by repulsions. At some points, VdW attractions and electrostatic repulsions can result in new arrangement of particles in strands. If salt concentration is well above CCC, the gelation might not occur and instead the dispersion forms a viscous suspension in which particle stranding is not present^9,11,26,27^. According to IUPAC terminology, sol–gel critical concentration is defined as “the concentration of an added electrolyte above which a particulate sol undergoes coagulation instead of gelation”^28^. As shown in Fig. 2, the gels turn into a viscous suspension at high salt concentrations beyond the sol–gel critical concentration.

**Figure 3:**
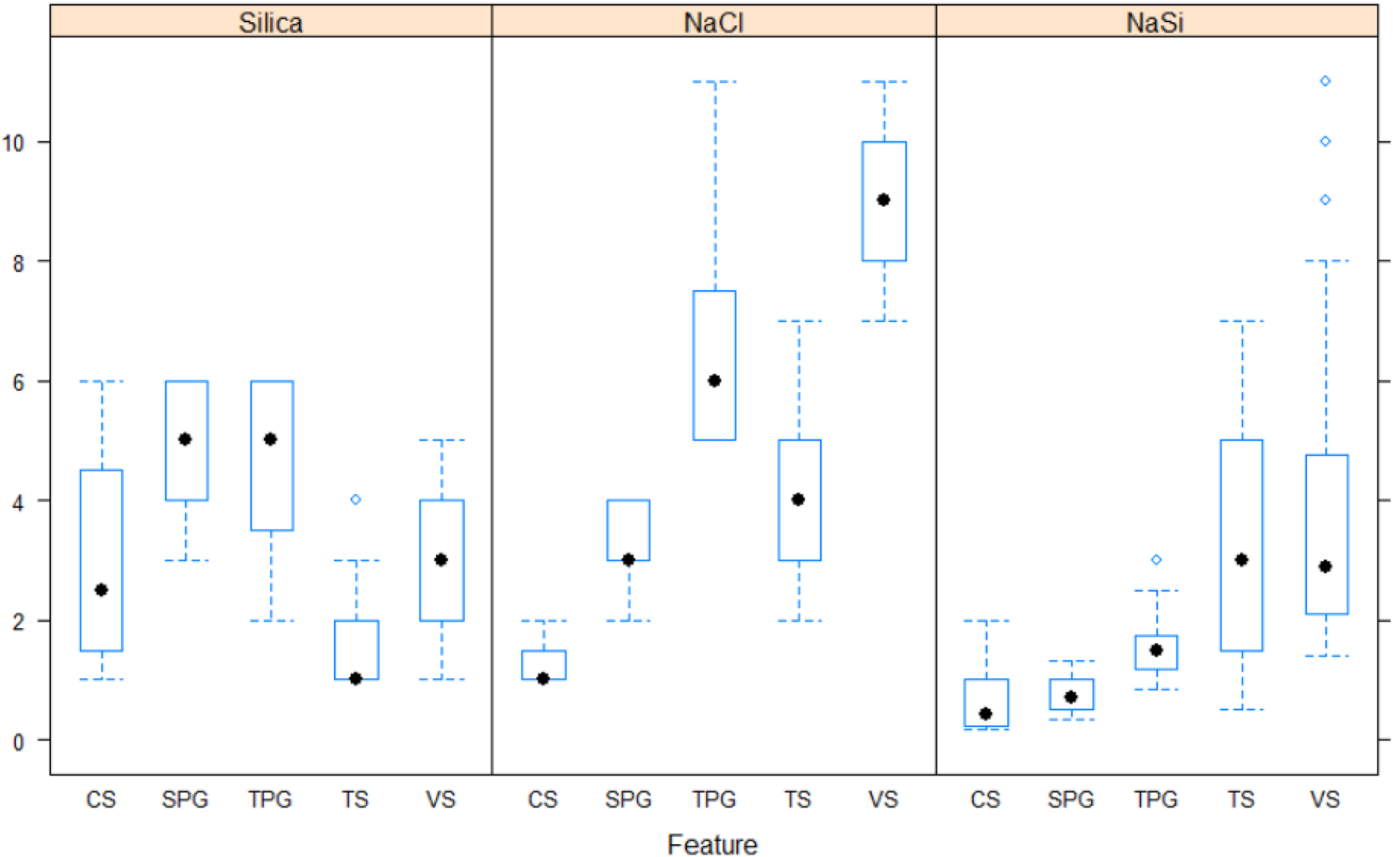
Median ± interquartile range (boxes) and 95% confidence intervals (whiskers) for silica, NaCl, and NaCl/Silica ratio based on different classes observed in bottle tests. *CS* clear suspension, *SPG* single-phase gel, *TPG* Two-phase gel, *TS* turbid suspension, *VS* viscous suspension.

**Figure 4:**
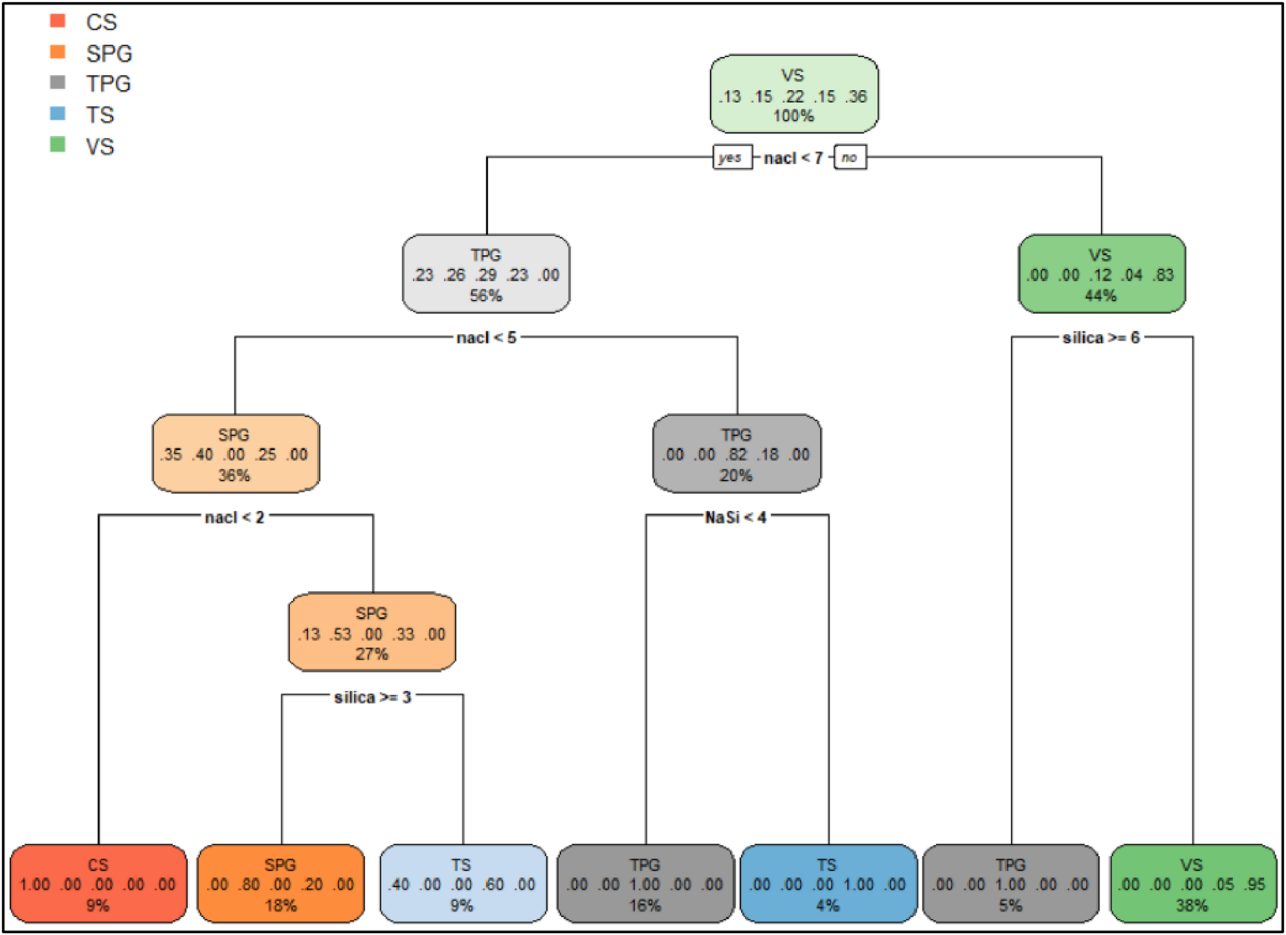
Tuned decision tree diagram developed by machine learning in R by CARET package^29^ using bottle test results. 10-k repeated cross validation with three repeats was performed using 20% of data (p = 0.8). Each node represents the predicted class (top), the predicted probability of each class (middle), the percentage of observations in the node (bottom). *CS* clear suspension, *SPG* single-phase gel, *TPG* Two-phase gel, *TS* turbid suspension, *VS* viscous liquid. Concentrations included on the nodes are in weight percentage.

Figure 3 shows the range of silica, NaCl, and NaCl/Silica ratio (NaSi) for different classes observed in bottle test results. As shown, the medians for different classes show a more meaningful trend for NaSi. Figure 4 shows the decision tree diagram developed with these three parameters (silica, NaCl, and NaSi) in R using machine learning by CARET^29^. The model was developed using 80% of data and cross validated by 20% of data using 10-k repeated cross-validation with three repeats. The diagram makes the decision regarding the fate of the dispersions after 24 h with an accuracy of 100% for the tuned model. Each node in Fig. 4 represents the predicted class (top), the predicted probability of each class (middle), the percentage of observations in the node (bottom). As can be seen, single- and two-phase gelation observations together are 39% of total observations and ranked first. This may outline the effect of NaCl on the gelation of bare silica.

### Gelation time

#### Time of complete gelation

**Figure 5:**
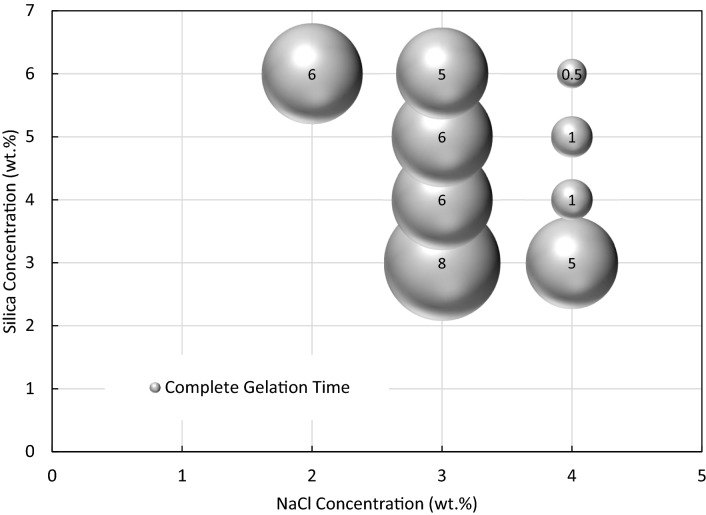
Bubble plot of gelation time (0.5–8.0 h) for different concentrations of silica (3–6 wt%) and NaCl (2–4 wt%) in DIW as gellants. The diagram represents the time required for a complete single-phase solid gelation obtained visually via bottle tests.

The gelation time of solid gels was first evaluated visually in bottle tests. Figure 5 shows the gelation time in hours for different concentrations of silica (3–6 wt%) and sodium chloride (3 and 4 wt%) in DIW. It should be noted that the gelation times here are the time of complete gelation spotted visually. This figure shows that an increase in salt concentration at a fixed silica particle concentration reduces the gelation time, which is more significant for particle concentrations ≥ 4 wt%. On the other hand, the gelation time decays with an increase in particle concentration at both salt concentrations (i.e., 3 and 4 wt%) following the power law (R^2^ > 0.91). The decreasing gelation time is more significant for the highest salt concentration. Therefore, the lowest complete gelation time was found with the gellant containing highest silica and NaCl concentrations in the window, i.e., 6 wt% and 4 wt% NaCl in DIW (top-right point).

#### Start time of gelation

**Figure 6:**
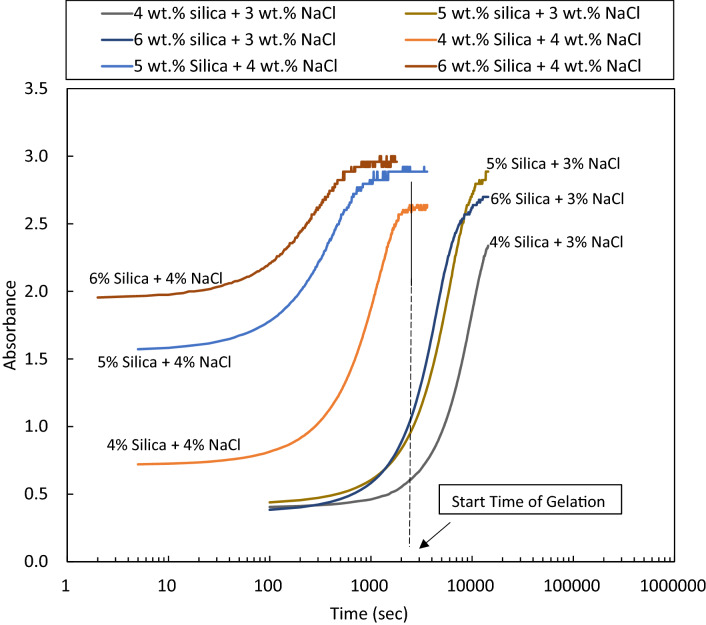
Absorbance versus time for the determination of start time of gelation for solid gels formed from different silica and NaCl concentrations in DIW.

**Figure 7:**
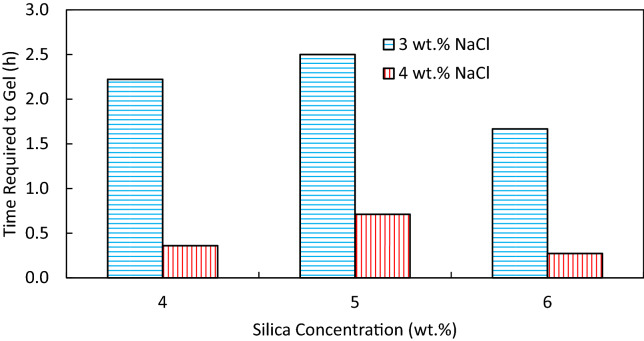
Time required to gel (complete gelation time by bottle tests—start time of gelation by absorbance measurements) for different solid gels formed from different concentrations of silica and NaCl in DIW.

The dispersions with final state of single-phase solid gel were selected for this analysis. The start time of gelation was determined by UV–Vis spectroscopy at 400 nm. Figure 6 shows the absorbance of different gellants at 400 nm with time. The first point at which the diagrams plateaued is considered as the start time of gelation. The difference between complete gelation time (determined by bottle tests) and start time of gelation (determined by absorbance measurements) enlightens the time required for each dispersion to gel completely. As shown in Fig. 7, reducing the salt concentration by 1 wt% at a fixed silica concentration significantly defers the time required to gel. The required time for gelation is relatively unchanged for a varying particle concentration at a given salt concentration.

### Effect of type of salt on gelation

To investigate the influence of type of cation on gelation, monovalent Na^+^ was replaced with divalent Mg^2+^ in few random dispersions. It is well documented that CCC is inversely proportional to the valence of the coagulating ion in the dispersion. Comparing NaCl and MgCl_2_, the higher valence cation can destabilize the dispersion more powerfully as the CCC is reduced by a factor of 64 when using Mg^2+^^30,31^. As can be seen from Fig. 2, it should be noted that gelation type is purely dependent on the salt concentration. In other words, increasing salt concentration at a fixed particle concentration does not necessarily gels the dispersions. As observed earlier, at high salt concentrations, viscous suspensions are created. Therefore, it is interesting to question how divalent cation affects different regions, especially the single-phase solid region, observed in Fig. 2.

**Figure 8:**
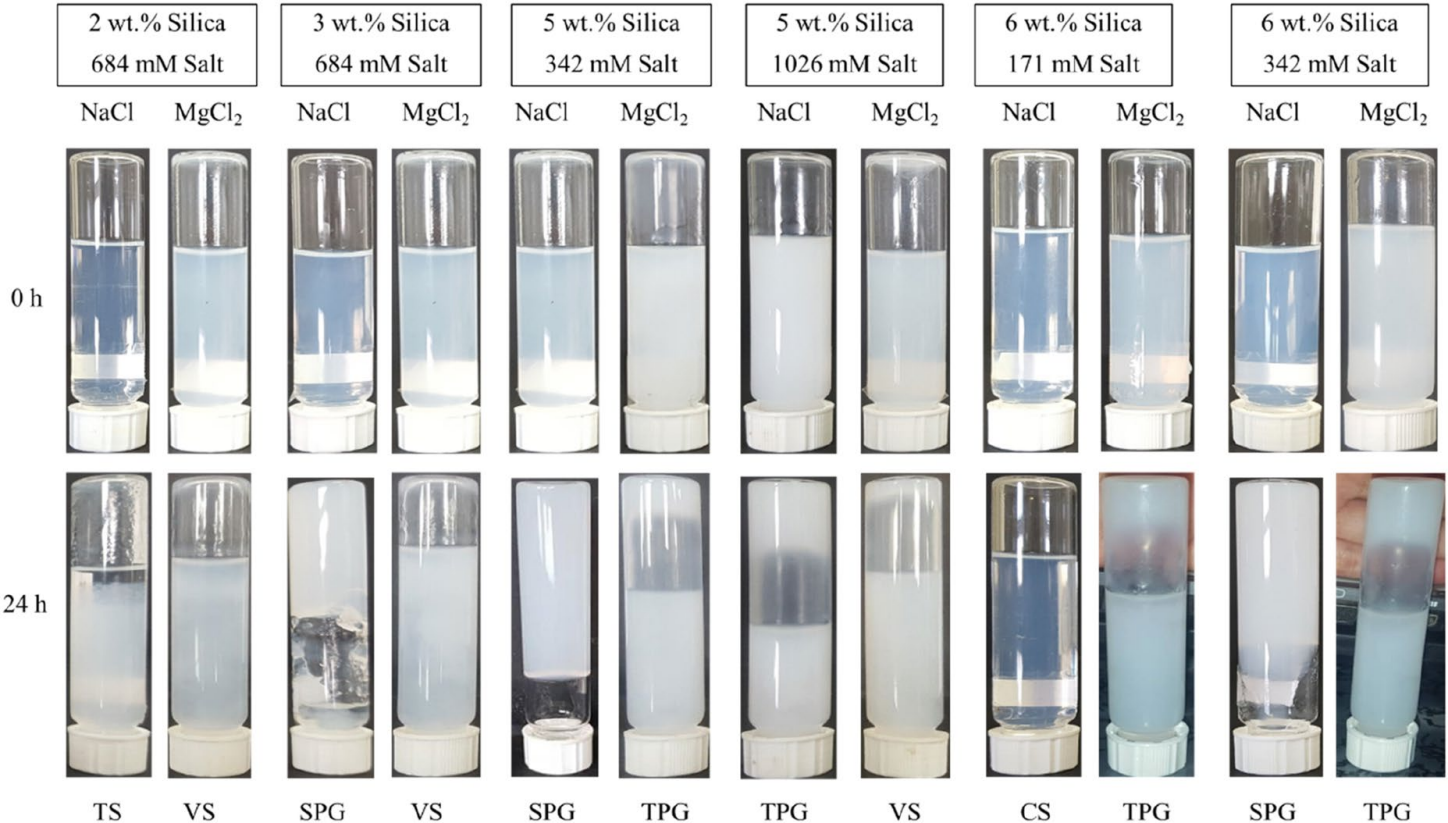
Effect of type of cation (Na^+^ or Mg^2+^) at different ionic strengths on the final status of the dispersions standing at room temperature of 20–25 °C after 24 h.

To have a proper comparison, ionic strengths were used. Figure 8 shows the results of the bottle tests for MgCl_2_ and NaCl. For instance, the dispersion containing 3 wt% silica and an ionic strength of 684 mM creates a single-phase solid gel by NaCl after 24 h while it is a viscous suspension with MgCl_2_. It is expected that for a single-phase solid gel, lower ionic strengths (100 mM) of MgCl_2_ are enough however a complete inspection of all lower/higher ionic strengths of MgCl_2_ is required to completely understand its effects.

### Gel characterization

#### Viscosity

Increasing the viscosity of injected fluid is an efficient way for the control of water conformance. Here, viscosities of single-phase solid gels were measured under various shear rates. Since shear rates near wellbore are normally between 10 and 100 s^–1^, the viscosities at these shear rates are of great importance^1^.

**Figure 9:**
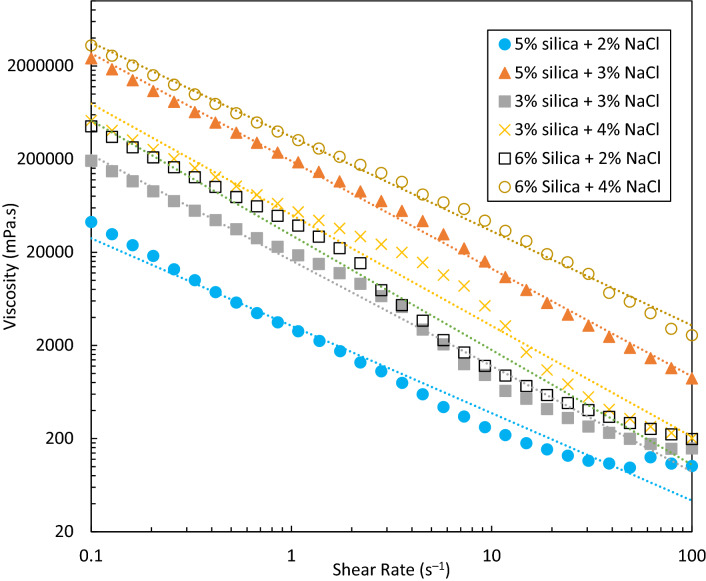
Viscosity versus shear rates for different single-phase solid gels formed by different dispersions at 25 °C. The test was performed after 24 h. The dotted lines show the power law fitted to each set of data points.

Figure 9 shows the viscosities of different gellants forming a single-phase solid gel after 24 h at different shear rates. It is observed that the viscosity is reduced by increasing the shear rate. The trend of viscosity with shear rates is approximately similar for all gellants, indicating a non-Newtonian behaviour (see Table S-1 in Supporting Information). The gel formed with 6 wt% silica and 4 wt% NaCl has the highest viscosity while the gel formed by 5 wt% silica and 2 wt% NaCl has the lowest viscosity at all shear rates. This minimum viscosity is considered enough to be used for water conformance control.

It is observed that at a fixed silica or NaCl concentration, increasing the other parameter concentration would increase the viscosity of final gels, indicating that both silica and NaCl concentrations are determining parameters, which agrees well with the results reported by Metin et al.^24^. The addition of only 1 wt% NaCl to the dispersion with 5 wt% silica and 2 wt% NaCl in DIW enhances the viscosity of the final gel dramatically. These findings suggest the higher importance of salt concentration on viscosity of gels.

#### Rheology

**Figure 10:**
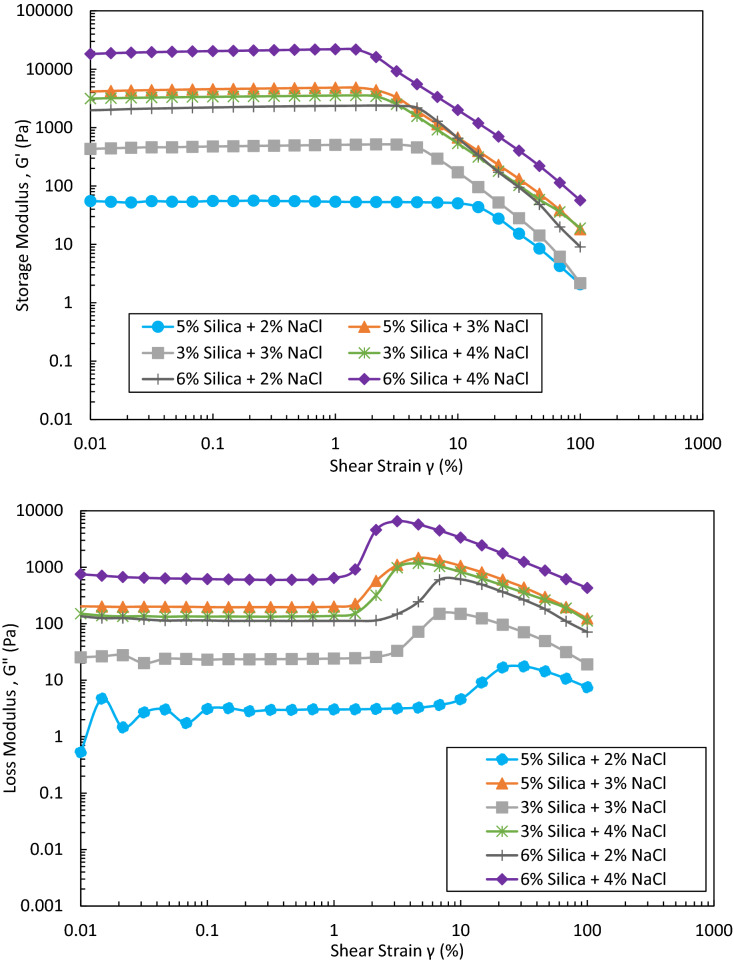
Storage modulus (G′) and loss modulus (G″) versus shear strain for different solid gels formed from different concentrations of silica and NaCl in DIW after 24 h.

Amplitude sweep method used during rheology analysis enables the evaluation of solid gel behaviours. Some single-phase solid gels observed in bottle tests were selected in this section to analyse the effect of particle/salt concentration on the rheology of the gels. Figure 10 shows the storage (G′) and loss moduli (G″) versus shear strains for different solid gels. As shown, increasing the silica concentration at a fixed salt concentration or vice versa increases storage and loss moduli at all shear strains. This increase is larger at high particle concentration and low salt concentration. The results obtained are similar to those reported previously^24^.

**Figure 11:**
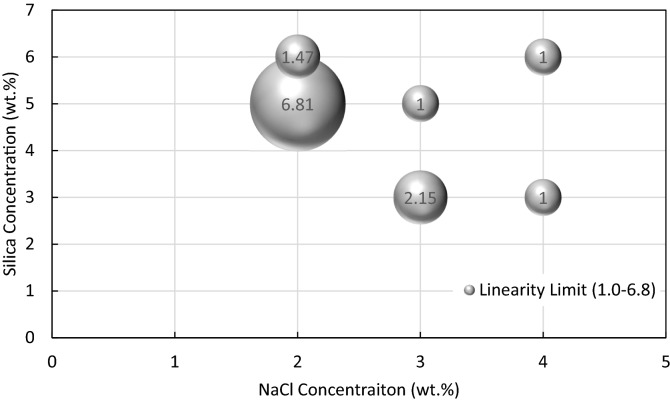
Bubble plot of limits of linear viscoelastic region as shear strain (%) for different solid gels formed from different concentrations of silica and NaCl in DIW at room temperature after 24 h.

The maximum shear strain applied on the gel with no breakdown of skeleton is called the limit of linear viscoelastic region. The linear viscoelastic region is usually determined as a window on G′ or G″ versus shear strain plot where points are on a straight line^32–34^. The limits of linear viscoelastic region of solid gels under study are plotted in Fig. 11. As shown, the gel formed by 5 wt% silica and 2 wt% NaCl has the largest elasticity. Post linear viscoelastic region can be used to study the creamy behaviour of the gels. G′ values of the linear viscoelastic region give some information about the strength of the gel. As shown in Fig. 10, the G′ diagrams experience a decrease with shear strain in the post linear viscoelastic region. The initial decreasing part is representative of the non-creamy structure of the gel with fragile breakdown while the following pure decreasing trend implies a gradual fracturing of the whole gel^34–36^.

Like G′, G″ diagrams initially plateaued in the linear viscoelastic region. The shear strain at which G″ starts increasing can be also considered as the limit of linearity (Fig. 10). The maximum G″ in the diagrams is related to the shear strain below which the gel is still solid-like. Beyond the maximum, G″ diagrams decrease with shear strains and the gel starts flowing. In the G″ rising region, G′ is higher than G″ i.e. the elastic behaviour of the viscoelastic gel is dominant. In this region, some bonds between particles in the gel network might be broken with shear strain but the gel is still solid-like with particles strongly saving the network. The loss of energy here is mainly due to the voids developed in the gel network because of small cracks which creates internal viscous friction with a convertibility into friction heat^33,37^. Beyond maximum G″, the breaking of the bonds in the particle network can be intensified such that the gel ruptures and starts flowing at higher shear strains i.e. the viscous behaviour of the viscoelastic gel would be dominant (Fig. 10). If the small cracks grow in the gel, they become bigger and bigger which manifests itself as a more viscous gel that tends to flow easily. In this case, G″ diagram becomes bigger than G′ diagram at different shear rates.

**Figure 12:**
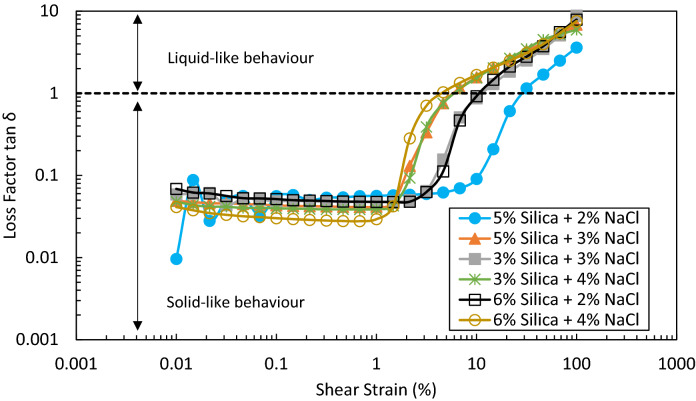
Loss factor (G″/G′) versus shear strain for solid gels formed from different concentrations of silica and NaCl in DIW after 24 h.

The yield point (i.e. soft point) and flow point of the gels can be determined using G′ or G″ versus shear stress (Fig. S-5 in Supporting Information). The yield point corresponds to the maximum shear stress below which the points are on a linear line and is equal to linearity limit. The flow point is the point at which G′ meets G″ (a loss factor of 1). Figure 12 illustrates a plot of loss factor (G″/G′) versus shear strain for different solid gels. As can be seen, where G″/G′ < 1, although there might be a reduction in the strength of the gel structure, it still behaves like a solid. On the other hand, in case G″/G′ > 1, the viscous part of the gel takes over and gel tends to flow easily. It is noteworthy to mention that in all gels under study, the flow point is equal to the yield point (Fig. S-5); thus, it may be inferred that the gels are more likely to fragile fracturing.

Gelation of colloidal particles causes elasticity while the particle network causes particle jamming/bridging and capillarity at interfaces. Gelation also brings about mechanical stability in the system. When finite elastic modulus in a colloidal dispersion appears, gelation occurs when attraction exceeds thermal energy which leads to the aggregation of particles and formation of a network of particles. This network could result in particle arrest (i.e. entrapment) and subsequent rheological features of the gel^38,39^.

### Displacement study

#### Control experiment

**Figure 13:**
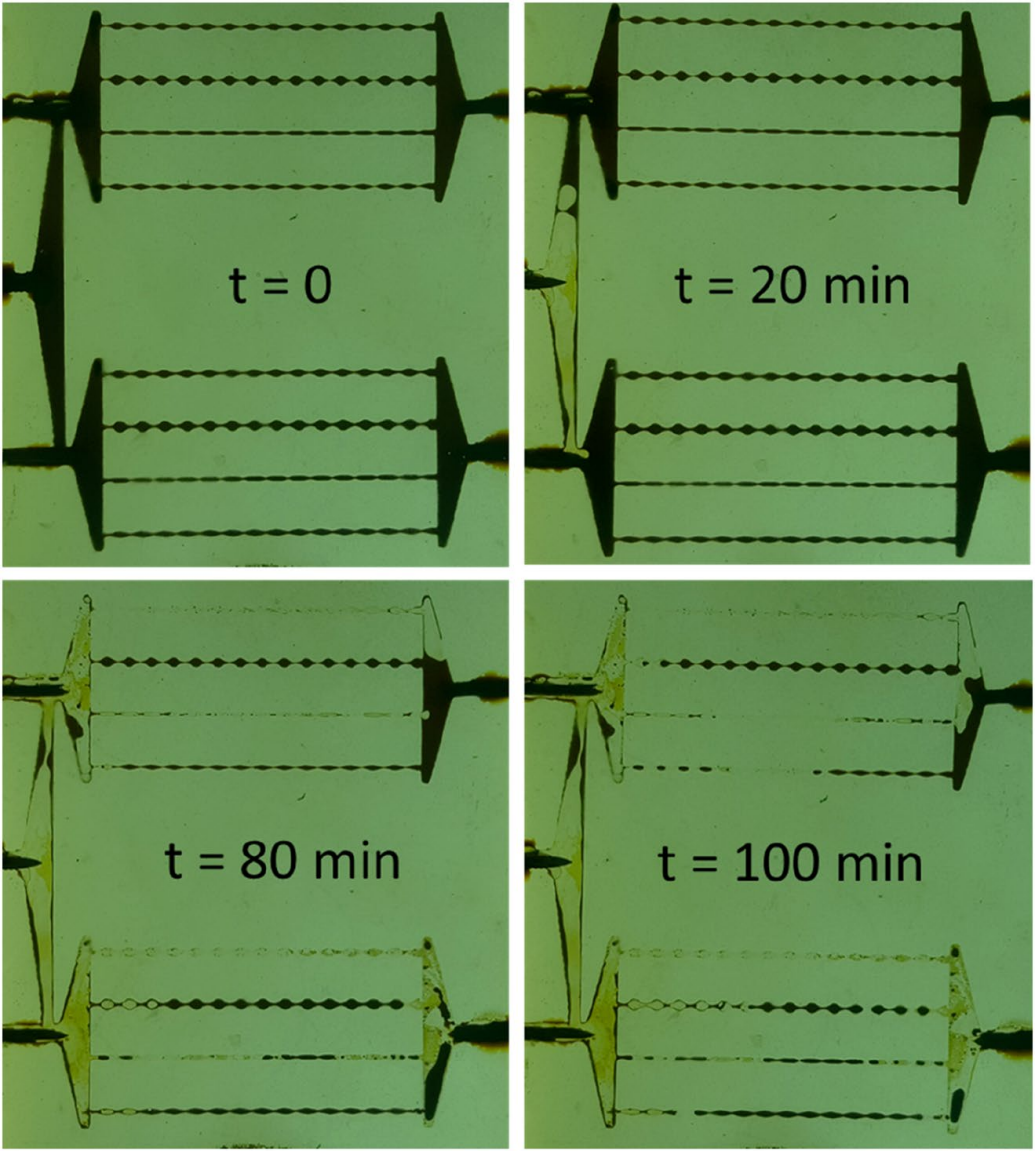
Control flooding experiment of DIW to a fully oil-saturated porous medium at different times. The letter “t” stands for time of flooding. The final photo corresponds to 2 PV injected DIW.

To ensure the normal performance of different parts of the designed glass micromodel, i.e. inlets and porous media, it was first fully saturated with crude oil and flooded with DIW. As can be seen from Fig. 13, DIW water has been injected through the main inlet into the two porous media for 2 PVs. Considering the final photo, it is observed that nearly the same amount of crude oil has been swept by DIW from the two porous media. The final oil recovery factor of two porous media is identically ~ 62% original oil in place in both porous media. These results confirm that the design of micromodel has been successful, and thus it is trustable for the main experiment.

#### Main experiment

Along with static investigations by rheology and viscosity measurements, the capability of the solid gels for water conformance control should be examined in porous medium. As the contact area between the gellant and porous media is higher, as compared to bottle tests, it is expected that the gels would have a higher strength in the porous medium. Jurinak and Summers declared that the strength of colloidal gel is dependent on the time taken after initial gelation^18^. Here, a fixed 24 h resting time as used for bottle tests was considered. The dispersion containing 3 wt% silica and 3 wt% NaCl was selected for this study. As can be seen from Fig. S-2, this dispersion forms a solid gel and is a boundary point close to turbid suspensions. According to the rheology and viscosity analyses, the solid gel formed by this dispersion does not provide the highest storage modulus/viscosity. Thus, if it successfully blocks the porous medium, we can claim that other stronger gels can be more effective.

**Figure 14:**
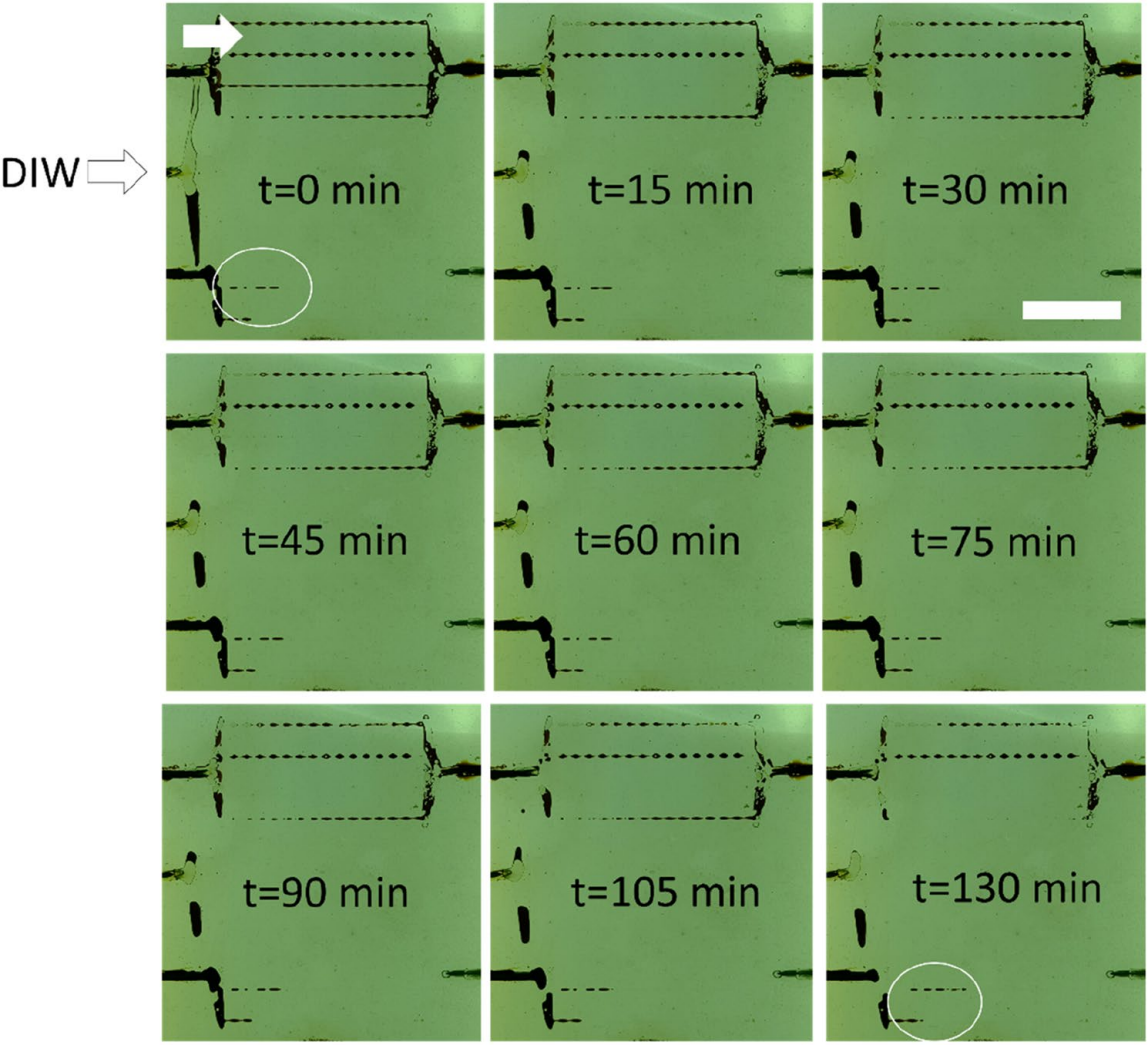
Macroscopic images of dual-patterned glass micromodel during injection of DIW for 2 PVs. The top and bottom porous media were initially saturated with crude oil and a solid gel formed from 3 wt% silica + 3 wt% NaCl in DIW after 24 h. The letter “t” shows the time of flooding. The white scale bar is 2 cm. The white arrow shows the flow direction.

As explained earlier, the bottom and top porous media of the glass micromodel were first saturated with the gellant and crude oil, respectively. Such experiment could mimic a water bearing zone topped by an oil layer which should be blocked by solid gels before the injection of an aqueous phase for potential enhanced oil recovery (EOR). The glass micromodel was allowed a resting time of 24 h for the dispersion to gel and for the oil to equilibrate with porous medium. The small amount of crude oil in the bottom porous medium encircled in white is related to the movement of oil from the oil-saturated porous media to the lower porous medium due to the pressure gradients induced by two fluids during resting time. DIW was injected into the glass micromodel through the main inlet while other inlets were blocked to prevent air entrance. As observed in Fig. 14, the dispersion in the bottom porous medium has strongly gelled after 24 h. It is expected that silica has been bonded to the glass surface through siloxane linkages to strongly block the porous medium. It is observed from the images that the small crude oil slug in the lower porous medium has not been displaced after 2 PV injection of DIW which itself proves that the gellant has been completely gelled to trap the oil. As well, over 2 PV injection, no liquid production was observed from the outlet connected to the bottom porous medium. During flooding, the solid gel was observed to divert the injected DIW toward the oil-saturated porous medium to recover oil. The dynamic oil sweeping from the top porous medium also confirms that formed gel in the bottom porous medium has not been displaced on applying pressure gradients by DIW which implies the suitability of the gel strength for water shut-off applications in oil reservoirs.

**Figure 15:**
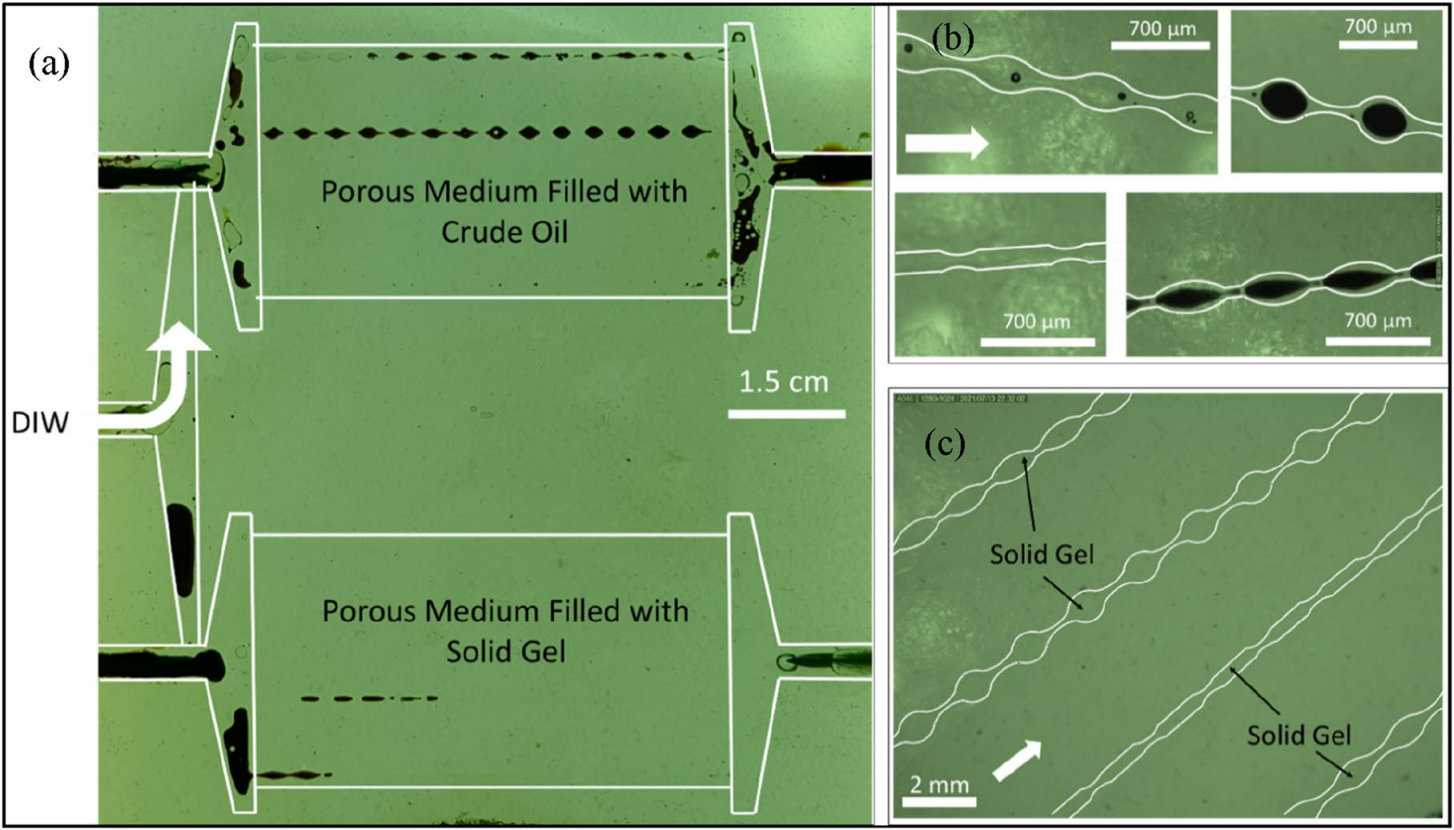
(**a**) Macroscopic image of the whole micromodel, (**b**) microscopic image of the top oil-saturated porous medium, and (**c**) microscopic image of the bottom gel-saturated porous medium. The images have been taken after 2 PV injection of DIW into the dual-patterned glass micromodel initially saturated with crude oil (top porous medium) and a solid gel formed from 3 wt% silica + 3 wt% NaCl in DIW after 24 h (bottom porous medium). The letter “t” shows the time of flooding. The white arrow shows the flow direction.

**Figure 16:**
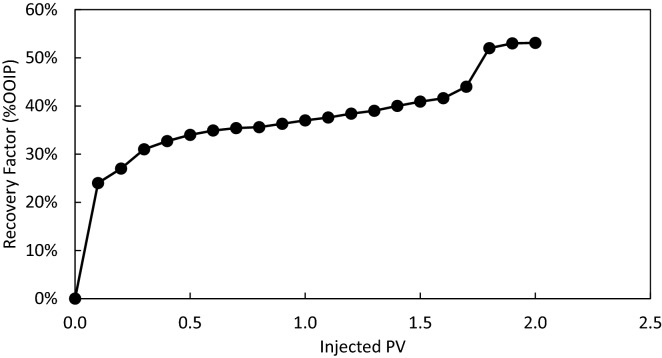
Ultimate oil recovery factor versus injected PV during injection of DIW into the dual patterned glass micromodel initially saturated with crude oil (top porous medium) and a solid gel formed from 3 wt% silica and 3 wt% NaCl in DIW (bottom porous medium).

Figure 15 shows the final macroscopic and microscopic images of glass micromodel after 2 PV injection of DIW. As expected, the top porous medium was not made oilwet during the resting time of 24 h thus the injected DIW has passed on the walls of the grains with oil left snapped in the middle of the pores. It is argued that the shape of pore throats and pore/throat aspect ratios have affected the extent of oil displaced from the tubes of the top porous medium. Figure 16 shows the oil recovery factor of the top porous medium versus the injected PV. The injected DIW caused a total oil recovery of 53% original oil in place which would not be possible without blockage of the bottom porous medium, i.e. water bearing zone, by the gel.

## Conclusions

Colloidal gels formed at room temperature by adding different concentrations of salt to hydrophilic silica dispersions were studied for water conformance control in oil reservoirs. A series of bottle tests were performed to characterize the gelation window regarding different particle and salt concentrations. The gelation time, effect of type of salt, rheology and viscosity were also inspected. The strength of the solid gel was then tested in porous media in a novel dual-patterned glass micromodel designed and constructed by the research group to mimic a water producer layer topped with oil layers. The key findings of this studies are summarized as follows:

- Bottle test results indicated a very well classified data with clear boundaries based on different silica (1–6 wt%) and NaCl (1–11 wt%) concentrations. Different classes were recognized after 24 h at room temperature: clear suspensions, single- or two-phase gels, and (non)viscous suspensions. It was concluded that single- or two-phase gelation is mostly dependent on the salt concentration, not particle concentration. The results showed that for a gel formation (single- or two-phase gelation), a high concentration of salt is not required. Higher NaCl concentrations at a constant silica concentration can indeed create viscous suspensions. Thus, the determination of gelation window for a specific salt is crucial in this regard. The tuned decision tree developed by machine learning can effectively predict the fate of dispersions after 24 h resting time at room temperature. 39% of total observations in bottle tests were single- and two-phase gels.
- Type of salt has a vital effect on the final state of dispersions. Monovalent sodium cation replacements with divalent magnesium at the same ionic strengths in some random dispersions showed a very different behaviour mainly due to the higher coagulating ability of divalent cation in colloids, which necessitates a complete study on MgCl_2_ as future work.
- The gelation times of solid gels were visually determined in bottle tests, denoted as complete gelation time, and further inspected through UV–Vis spectroscopy, denoted as start time of gelation. The difference between these two times provided good estimates over the required time for each gellant to gel completely. In the single-phase solid gelation window, the highest silica and NaCl concentrations owned the smallest gelation time i.e. 6 wt% silica and 4 wt% NaCl in DIW. An additional 1 wt% salt reduced the gelation time by 2.5 times.
- Rheology and viscosity analyses agreed well with gelation time results with the same parametric dependence on particle and salt concentrations. At high shear rates i.e. 100 s^–1^, the formed solid gels showed a viscosity, storage modulus and loss modulus of 100–2600 mPa s, 2–57 Pa and 7–430 Pa respectively which are high enough to withstand pressure gradient applied in the wells. The gel formed by 5 wt% silica and 2 wt% NaCl has the largest elasticity. In single-phase solid gelation window, the gel with shortest gelation time had the highest viscosity and storage/loss modulus at all shear strains. However, this gel was not the most elastic solid gel, based on limits of linear viscoelastic regions. Strong gels can be fragile on applying shear stresses and should be avoided for water conformance controls.
- Injection of DIW into a dual-patterned glass micromodel initially saturated with crude oil in top porous medium and a solid gel in bottom porous medium showed an efficient performance of the solid gel in blocking the bottom porous medium for the injected DIW to divert its flow toward the oil-saturated zone for potential EOR. A total oil recovery of 53% original oil in place was achieved which would not be possible without blockage of the bottom porous medium i.e. water bearing zone by the gel. To the best knowledge of the authors, such a promising observation in a visualization study to prove the applicability of colloidal gels for water shut-off applications has not been addressed yet.
- Other parameters such as temperature, type of ion, particle size, and particle surface wettability are potentially important in gelation which must be considered in future work.

## Supplementary Information

Supplementary Information.

## References

[1] Sydansk RD, Romero-Zerón L (2011). Reservoir Conformance Improvement.

[2] Afi F (2021). SPE/IATMI Asia Pacific Oil & Gas Conference and Exhibition.

[3] Bai B, Zhou J, Yin M (2015). A comprehensive review of polyacrylamide polymer gels for conformance control. Pet. Explor. Dev..

[4] Yu L, Dong M, Ding B, Yuan Y (2018). Experimental study on the effect of interfacial tension on the conformance control of oil-in-water emulsions in heterogeneous oil sands reservoirs. Chem. Eng. Sci..

[5] Ding B, Dong M (2019). Optimization of plugging high mobility zones in oil sands by injection of oil-in-water emulsion: Experimental and modeling study. Fuel.

[6] Rezvani H (2020). A novel foam formulation by Al2O3/SiO2 nanoparticles for EOR applications: A mechanistic study. J. Mol. Liq..

[7] Li X, Nakagawa S, Tsuji Y, Watanabe N, Shibayama M (2019). Polymer gel with a flexible and highly ordered three-dimensional network synthesized via bond percolation. Sci. Adv..

[8] Partlow DP, Yoldas BE (1981). Colloidal versus polymer gels and monolithic transformation in glass-forming systems. J. Non-Cryst. Solids.

[9] Rouwhorst J, Ness C, Stoyanov S, Zaccone A, Schall P (2020). Nonequilibrium continuous phase transition in colloidal gelation with short-range attraction. Nat. Commun..

[10] Dibble CJ, Kogan M, Solomon MJ (2008). Structural origins of dynamical heterogeneity in colloidal gels. Phys. Rev. E.

[11] Lu PJ (2008). Gelation of particles with short-range attraction. Nature.

[12] Trappe V, Prasad V, Cipelletti L, Segre P, Weitz DA (2001). Jamming phase diagram for attractive particles. Nature.

[13] Puertas AM, Odriozola G (2007). Linking phase behavior and reversible colloidal aggregation at low concentrations: Simulations and stochastic mean field theory. J. Phys. Chem. B.

[14] Moghimi E, Schofield AB, Petekidis G (2021). Yielding and resolidification of colloidal gels under constant stress. J. Phys. Condens. Matter..

[15] Campbell AI, Anderson VJ, van Duijneveldt JS, Bartlett P (2005). Dynamical arrest in attractive colloids: The effect of long-range repulsion. Phys. Rev. Lett..

[16] Manley S (2005). Time-dependent strength of colloidal gels. Phys. Rev. Lett..

[17] Smith WE, Zukoski CF (2006). Aggregation and gelation kinetics of fumed silica–ethanol suspensions. J. Colloid Interface Sci..

[18] Jurinak J, Summers L (1991). Oilfield applications of colloidal silica gel. SPE Prod. Eng..

[19] Liang J, Sun H, Seright R (2022). SPE/DOE Enhanced Oil Recovery Symposium.

[20] Dai C, You Q, He L, Zhao F (2011). Study and field application of a profile control agent in a high temperature and high salinity reservoir. Energy Sources A.

[21] Lakshtanov DL, Sinogeikin SV, Bass JD (2007). High-temperature phase transitions and elasticity of silica polymorphs. Phys. Chem. Miner..

[22] Lowry T (1929). The properties of silica. Nature.

[23] Sögaard C, Funehag J, Abbas Z (2018). Silica sol as grouting material: a physio-chemical analysis. Nano Converg..

[24] Metin CO, Rankin KM, Nguyen QP (2014). Phase behavior and rheological characterization of silica nanoparticle gel. Appl. Nanosci..

[25] Puertas AM, Fuchs M, Cates ME (2003). Simulation study of nonergodicity transitions: Gelation in colloidal systems with short-range attractions. Phys. Rev. E.

[26] Zaccarelli E (2005). Model for reversible colloidal gelation. Phys. Rev. Lett..

[27] Zaccarelli E (2007). Colloidal gels: equilibrium and non-equilibrium routes. J. Phys. Condens. Matter.

[28] Alemán JV (2007). Definitions of terms relating to the structure and processing of sols, gels, networks, and inorganic-organic hybrid materials (IUPAC Recommendations 2007). Pure Appl. Chem..

[29] Kuhn M (2008). Building predictive models in R using the caret package. J. Stat. Softw..

[30] Hardy W (2002). A preliminary investigation of the conditions which determine the stability of irreversible hydrosols. J. Phys. Chem..

[31] Schulze H (1882). Schwefelarsen in wässriger Lösung. J. Prakt. Chem..

[32] Shah S, Chen Y-L, Schweizer K, Zukoski C (2003). Viscoelasticity and rheology of depletion flocculated gels and fluids. J. Chem. Phys..

[33] Rueb C, Zukoski C (1997). Viscoelastic properties of colloidal gels. J. Rheol..

[34] Mezger T (2020). The Rheology Handbook.

[35] Nabizadeh M, Jamali S (2021). Life and death of colloidal bonds control the rate-dependent rheology of gels. Nat. Commun..

[36] Tsurusawa H, Leocmach M, Russo J, Tanaka H (2019). Direct link between mechanical stability in gels and percolation of isostatic particles. Sci. Adv..

[37] Pal A (2020). Anisotropic dynamics and kinetic arrest of dense colloidal ellipsoids in the presence of an external field studied by differential dynamic microscopy. Sci. Adv..

[38] Amiri A, Øye G, Sjöblom J (2011). Temperature and pressure effects on stability and gelation properties of silica suspensions. Colloids Surf. A.

[39] Whitaker KA (2019). Colloidal gel elasticity arises from the packing of locally glassy clusters. Nat. Commun..

