# Supplementary material for: Experimental characterization of colloidal silica gel for water conformance control in oil reservoirs
Source: Sci Rep. 2022 Jun 10;12:9628. doi: 10.1038/s41598-022-13035-1 (PMC9187666; doi:10.1038/s41598-022-13035-1)
Supplement: Supplementary file 1 — Supplementary Information. [file 41598_2022_13035_MOESM1_ESM.docx]

**Experimental Characterization of Colloidal Silica Gel for Water Conformance Control in Oil Reservoirs**

Zahra Ghaffari^a,b^, Hosein Rezvani^a,c^, Ali Khalilnezhad^a,b^, Farid B. Cortes^d^, Masoud Riazi^a,e*^

^a^ Enhanced Oil Recovery (EOR) Research Centre, IOR/EOR Research Institute, Shiraz University, Shiraz, Iran

^b^ Faculty of Petroleum and Natural Gas Engineering, Sahand University of Technology, Tabriz, Iran

^c^ Department of Chemistry, University of Hull, Hull, UK

^d^ Grupo de Investigación en Fenómenos de Superficie-Michael Polanyi, Departamento de Procesos y Energía, Facultad de Minas, Universidad Nacional de Colombia, Sede Medellín, Medellín 050034, Colombia

^e^ Department of Petroleum Engineering, School of Chemical and Petroleum Engineering, Shiraz University, Shiraz, Iran

* Corresponding author:

Masoud Riazi (MR)

Email: [mriazi@shirazu.ac.ir](mailto:mriazi@shirazu.ac.ir)

**Supporting Information**

This supporting information contains some basic results.

Before doing bottle tests, as a control, different concentrations of silica (1–6 wt.%) in DIW (no salt) were prepared from the stock dispersion and monitored for 24h at room temperature. Figure S-1 shows the appearance of dispersions after 24h in which no trace of turbidity, sedimentation, or gelation of silica is observed when NaCl salt is absent. As illustrated, all dispersions are blueish, as they looked initially. The figure confirms that particles alone (no salt) cannot form a gel at this concentration range, and the gelation is purely related to the effect of added ions on the interactions between particles.


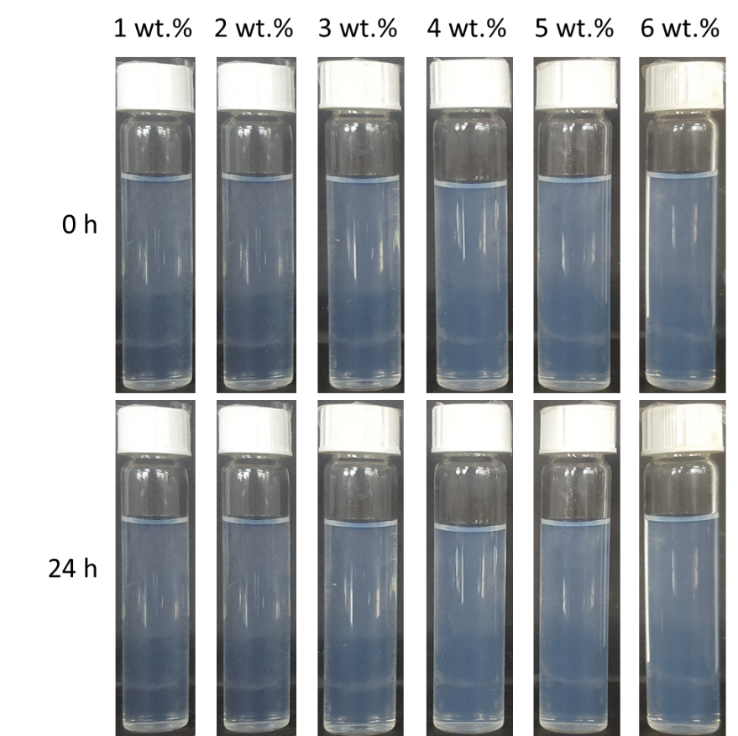


**Figure S-1.** Different concentrations of silica (1–6 wt.%) in DIW (no salt) diluted from the stock dispersion.

Figure S-2 shows the fate of different dispersions containing different concentrations of silica and NaCl in DIW placed at room temperature for 24h. The figure shows very well classified data. Figure S-3 shows a typical photo of different classes obtained after 24h in glass vials.


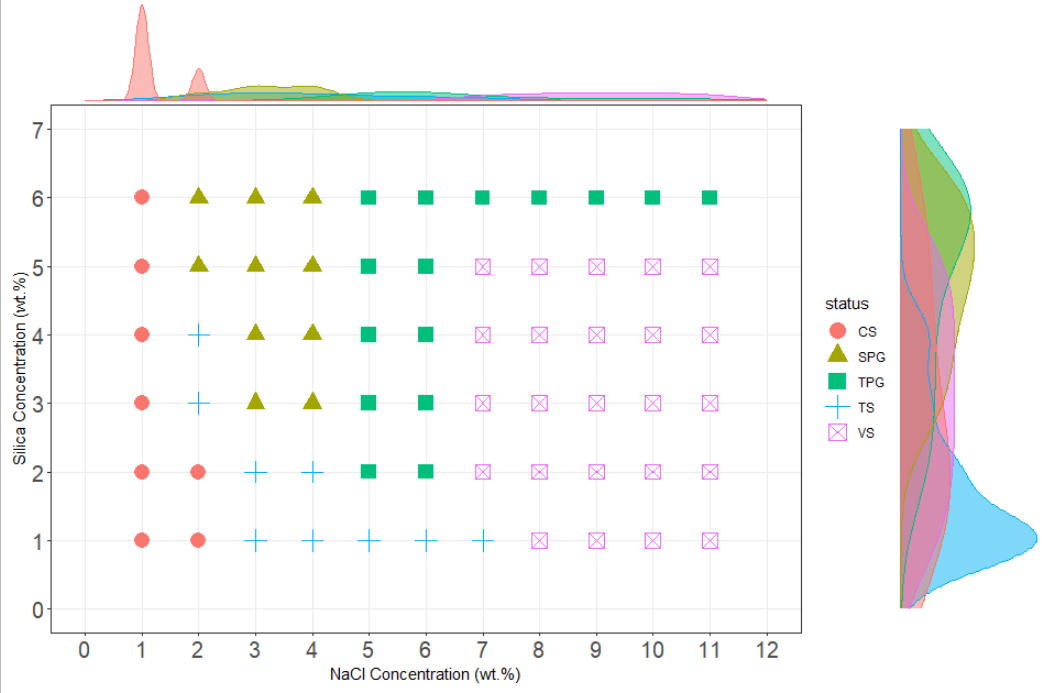


**Figure S-2.** Status of different dispersions containing different concentrations of silica and sodium chloride concentrations in DIW at room temperature of 20-25°C after 24h (pH=7–10). The outer colored diagrams show the density plots of each class relative to the opposite x- or y-axis. CS: clear suspension, SPG: single-phase gel, TPG: Two-phase gel, TS: turbid suspension, and VS: viscous suspension.


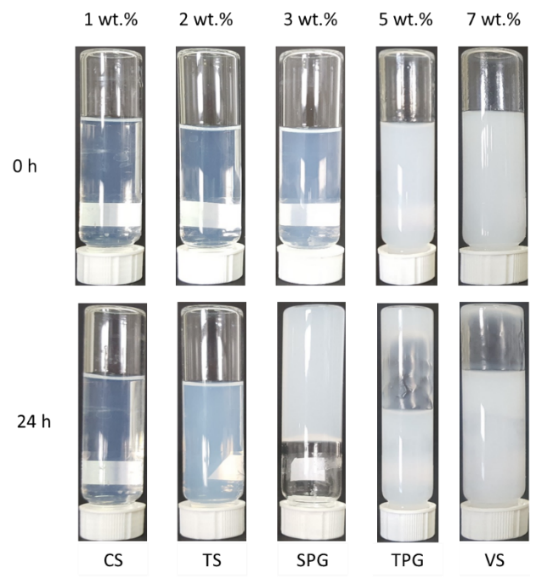


**Figure S-3.** Status of different dispersions containing a fixed 3 wt.% silica and different concentrations of sodium chloride in DIW at room temperature of 20-25°C after 24h. CS: clear suspension, SPG: single-phase gel, TPG: Two-phase gel, TS: turbid suspension, and VS: viscous suspension.

The start time of gelation was determined by UV-vis spectroscopy. Figure S-4a shows the full absorbance spectrum (400–1000 nm) of different concentrations of silica (3–6 wt.%) in DIW (no salt). The plot has been calibrated with DIW (no particle) as background. Different wavelengths from the full spectrum were selected to determine the best wavelength with the highest R-squared of the fitted lines. As can be seen from Figure S-4b, the highest accuracy (R^2^ = 0.96) is associated with 400 nm, which was selected for the main experiment i.e., determination of start time of gelation.

**Figure S-4.** (a) Full absorbance spectrum (400–1000 nm) and (b) Absorbance at wavelengths of 400, 450, 500, and 600 nm related to fresh dispersions containing different concentrations of silica in DIW (no salt) for the determination of candidate wavelength with the highest accuracy (i.e. highest R^2^).

The measured viscosities of gels with different concentrations of silica and NaCl at different shear rates show that the fluids behave like non-Newtonian fluids. The viscosity of non-Newtonian fluids could be related to the shear rate as follows:

$\mu_{eff.}=K \gamma^{n-1}$ (1)

where *µ_eff._* represents the effective viscosity (Pa.s), *K* is the flow consistency index and equals the viscosity at the shear rate 1 s^-1^, *γ* is the shear rate and *n* is the flow behavior index [1]. The calculated *n* and *K* values of the solid gels under study are represented in Table S-1. The higher the *K* value, the greater the viscosity of the gel.

**Table S-1.** Power law parameters for viscosities of different single-phase solid gels formed by different dispersions at 25°C. The test was carried out after 24h.

| Silica Concentration  (wt.%) | NaCl Concentration  (wt.%) | K  (Pa sn) | n  (–) | R^2^  (frc.) |
| --- | --- | --- | --- | --- |
| 5 | 2 | 3252.5 | 0.062 | 0.9869 |
| 5 | 3 | 190620 | -0.156 | 0.9995 |
| 3 | 3 | 16311 | -0.133 | 0.9972 |
| 3 | 4 | 50522 | -0.192 | 0.9908 |
| 6 | 2 | 30484 | -0.232 | 0.9939 |
| 6 | 4 | 347608 | -0.013 | 0.9998 |

Figure S-5 shows the storage and loss moduli of different gellants at different shear stresses. The yield point (i.e. soft point) and flow point of the gels can be determined using this plot. The yield point corresponds to the maximum shear stress below which the points are on a linear line and is equal to the linearity limit. The flow point is the point at which G′ meets G″ (a loss factor of 1). It is noteworthy to mention that in all gels understudy, the flow point is equal to the yield point.

**Figure S-5.** Storage modulus (G′) and loss modulus (G″) versus shear stress for different solid gels formed from different concentrations of silica and NaCl in DIW after 24h.

**References**

[1] R.B. Bird, Annual Review of Fluid Mechanics 8 (1976) 13.
